# Understanding the Pathophysiology of Chronic Pancreatitis: A Comprehensive Review Unraveling Pain Mechanisms and the Role of Psychosocial Factors

**DOI:** 10.3390/jcm15103831

**Published:** 2026-05-15

**Authors:** Aadhi C. Sekhar, Suganya Kandhi, Padmavathi Ramaswamy, Mohanapriya Thyagarajan, Manikya Kuriti, Appakalai N. Balamurugan

**Affiliations:** 1 Sri Ramachandra Medical College and Research Institute, Sri Ramachandra Institute of Higher Education and Research (SRIHER), Porur, Chennai 600116, Tamil Nadu, India; m0123171@sriher.edu.in; 2 Department of Physiology, Sri Ramachandra Medical College and Research Institute, Sri Ramachandra Institute of Higher Education and Research (SRIHER), Porur, Chennai 600116, Tamil Nadu, India; 3 The Controller of Examinations (CoE), Sri Ramachandra Institute of Higher Education and Research (SRIHER), Porur, Chennai 600116, Tamil Nadu, India; rpadmavathi@sriramachandra.edu.in; 4 Department of Surgery, Sri Ramachandra Medical College and Research Institute, Sri Ramachandra Institute of Higher Education and Research (SRIHER), Porur, Chennai 600116, Tamil Nadu, India; mohanapriyadr@gmail.com; 5 Wendy Novak Diabetes Institute, Norton Children’s Research Institute, Norton Healthcare, Louisville, KY 40202, USA; manikya.kuriti@nortonhealthcare.org (M.K.); bala.appakalai@louisville.edu (A.N.B.); 6 Division of Endocrinology, Department of Pediatrics, Pediatric Research Institute, University of Louisville, Louisville, KY 40202, USA

**Keywords:** chronic pancreatitis, pain and anxiety, SDGs (Sustainable Development Goals), quality of life, fibrosis, inflammation, TPIAT

## Abstract

Chronic pancreatitis (CP) is a fibro-inflammatory condition defined by permanent anatomical changes in the pancreas. The causes of CP are described by the TIGAR-O classification system: toxin-related, idiopathic, genetic mutations, autoimmune disorders, episodes of recurrent acute pancreatitis, and obstructions. Pain is multifactorial in nature, and common psychopathological consequences of CP, including depression and anxiety, complicate the clinical picture of chronic pancreatitis. As a result, the quality of life of patients with CP is decreased. This review describes the pathophysiology of pain and its relationship to underlying psychological consequences, with a focus on a long-term, holistic management approach. Strategies that combine physical and psychological management align with SDG 3 (Good Health and Well-being). CP predominantly affects patients from low socioeconomic backgrounds due to disparities in medical care, underscoring the relevance of achieving SDG 10 (Reduced Inequalities). This review emphasizes the importance of targeted research in developing a holistic care model for CP that aligns with the SDGs.

## 1. Chronic Pancreatitis (CP)

CP is a condition characterized by multiple components that result in recurrent pancreatitis and eventual fibrosis. Consequently, endocrine and exocrine deficiencies often arise [1]. The TIGAR-O system classifies risk factors into toxins, idiopathic causes, genetic mutations, autoimmune diseases, recurring and severe episodes of acute pancreatitis, and obstructive causes [2].

In adults, the main causes of CP are alcohol consumption, autoimmunity, and genetic mutations [3]. The etiology of pediatric CP does not follow the same pattern, as it is rooted predominantly in genetic mutations rather than lifestyle factors. In this population, genetic risk factors, such as mutations in cationic trypsinogen (PRSS1), cystic fibrosis transmembrane conductance regulator (CFTR), chymotrypsin C (CTRC), carboxypeptidase 1 (CPA1), and the pancreatic secretory trypsin inhibitor genes, are the major contributors to this condition. Non-genetic risk factors include obstructions, trauma, infections, and metabolic abnormalities. Traditional management of painful chronic pancreatitis follows conservative medical therapy, followed by invasive interventions. Initial strategies focus on pharmacological control using non-opioid analgesics, neuromodulators for nerve pain, and pancreatic enzyme replacement (PERT) to reduce organ workload. When pain persists, clinicians employ interventional techniques such as celiac plexus blocks to interrupt pain signaling or endoscopic procedures (ERCPs) and lithotripsy to clear ductal obstructions. Surgery, the final step, remains reserved for severe cases to provide permanent drainage or remove damaged tissue. These often fail to provide long-lasting effects. When pain becomes refractory, a total pancreatectomy (TP) may be performed. This procedure removes the entire organ, eliminating both insulin and glucagon secretion, leaving patients prone to brittle diabetes and recurrent severe hypoglycemic events (SHEs) due to the loss of pancreatic counter-regulatory function [4,5,6,7,8]. In children, pancreatitis does not resolve or improve; in fact, it **gets** worse over time and leads to permanent damage [9].

The major complaint of patients with this condition is persistent epigastric pain. The pain usually presents as a sharp, stabbing sensation or dull ache radiating to the back. This is usually aggravated by the intake of food and fluids [10]. The associated complaints usually include nausea, vomiting, and weight loss. Painful episodes can be related to the intake of fatty or high-fiber foods, consumption of alcohol, or extreme states of stress [11].

Chronic pain has been linked to psychological disturbances such as anxiety. This is caused by a vicious cycle in which anxiety increases levels of pain, and, in turn, increases anxiety levels. Patients’ anticipation of future painful episodes and fear of the progression of their illness increase their anxiety levels [12]. Anxiety is known to alter sleep patterns and activate stress responses, leading to an overall decrease in a patient’s quality of life [13]. This review focuses on the development of pain through a critical analysis of literature, examining its relation with anxiety.

## 2. Pathophysiology and Progression of Pain in Chronic Pancreatitis

The pain mechanisms in CP include inflammation, fibrosis, ductal obstruction, neurogenic inflammation, visceral hypersensitivity, and central sensitization. The pain associated with CP varies in nature depending on the duration since onset. In the initial stages, pain is intermittent and associated with inflammation. During subsequent stages, it becomes severe and loses the correlation with the inflammatory process in the pancreas [1,2,10]. This is not due to pancreatic damage but rather changes in the pain mechanism because of inflammation.

The pathogenesis of pain in CP involves the neuroimmune axis characterized by pathological remodeling of the peripheral and central nervous systems [14]. Chronic inflammatory stimuli activate pancreatic stellate cells (PSCs), which secrete nerve growth factor (NGF) and brain-derived neurotrophic factor (BDNF), driving neural hypertrophy and increased intrapancreatic nerve density [15,16]. This neuroplasticity is exacerbated by the infiltration of mast cells and macrophages, which secrete a pro-nociceptive secretome modulating the activation kinetics of Protease-Activated Receptor 2 (PAR2) and Transient Receptor Potential Vanilloid 1 (TRPV1) channels on primary afferent fibers [12,17]. The persistent release of neuropeptides like Substance P and CGRP induces neurogenic inflammation, culminating in peripheral sensitization [14]. Ultimately, the sustained nociceptive barrage triggers central sensitization in the dorsal horn of the spinal cord, manifesting as generalized hyperalgesia and persistent pain that often becomes independent of the initial injury to the gland [14,18].

The pathogenesis and psychosocial landscape of chronic pancreatitis (CP) are defined by profound etiological heterogeneity, where the primary insult dictates distinct pathophysiological trajectories and patient experiences. Clinically, alcohol-induced CP typically involves accelerated fibrogenesis and toxic-metabolic stress, often accompanied by systemic inflammation and nutritional comorbidities. In contrast, autoimmune pancreatitis (AIP) represents a distinct fibroinflammatory phenotype, frequently presenting with obstructive icterus and high sensitivity to corticosteroid therapy [19]. In the pediatric and hereditary cohorts, the disease trajectory is initiated by congenital pathogenic variants (e.g., PRSS1, SPINK1, and CFTR), which establish premature neural remodeling and prolonged nociceptive exposure [20,21].

These distinct biological pathways facilitate divergent psychosocial phenotypes. Patients with alcohol-related disease frequently contend with a psychological burden that correlates with an increased prevalence of major depressive disorder and substance-use sequelae [14]. Conversely, pediatric and genetic populations exhibit significant developmental dysregulation, where chronic pain leads to school absenteeism, social withdrawal, and the early establishment of pain catastrophizing [22]. Across all age groups, severe abdominal pain remains the most significant driver of reduced Health-Related Quality of Life (HRQoL), as the sustained nociceptive barrage ultimately triggers central sensitization, rendering the pain state autonomous and often independent of the initial glandular injury [14,22].

Quantitative tests on individuals showed that the pain threshold decreased not only in the abdominal region but also in areas distant from the pain. This shows generalized nervous system sensitization [10,23].

During the initial phase of the disease, pain is typically episodic and arises from acute inflammation and ductal obstruction, leading to the release of inflammatory mediators, including prostaglandins and cytokines, that activate nociceptive signaling pathways [1,2,10]. The pathogenesis of pain in chronic pancreatitis (CP) is an overlapping neurobiological continuum where peripheral mechanical triggers and central maladaptive changes occur in tandem rather than in a linear sequence. These acute pain episodes are often linked to eating, suggesting that pancreatic stimulation may induce intraductal hypertension, sensitizing pancreatic nociceptors to these inflammatory mediators and leading to peripheral sensitization; these nerves then undergo neural remodeling, enhancing pain sensitivity [10]. There is also increased activation of dorsal horn neurons and decreased inhibition of descending pain pathways, leading to central sensitization [24]. The pain signaling persists due to the pancreatic damage and fibrosis caused by recurrent inflammation, which can compress the intrapancreatic nerves and lead to neuropathic pain, including burning sensations, paresthesia, and allodynia [11,25]. In addition, ischemic pain may occur due to calcifications and ductal strictures, which can exacerbate discomfort [26]. Recurrent inflammatory injury results in neurogenic inflammation of the pancreas. The activation of pancreatic afferent nerves leads to the release of substance P and calcitonin gene-related peptide (CGRP), exacerbating inflammatory responses and activating nociceptors. Over time, the dorsal horn undergoes structural and functional changes due to continuous afferent signaling. The central sensitization involves the conversion of transient nociceptive inputs into a state of sustained neuronal hyperexcitability. Recent investigations have described abnormal descending inhibitory modulation in CP patients, indicating that descending pain-inhibitory controls have become abnormal and ineffective [24]. Neuroimaging investigations demonstrate decreased cortical thickness in regions important for pain processing, including the anterior cingulate cortex and insula, indicating significant pain-related neuromatrix remodeling in patients with CP [27]. These changes may explain the reason for continuous and severe pain despite unremarkable imaging. A profound dissociation exists between structural disease and the clinical pain phenotype in CP, where imaging severity frequently fails to predict the patient’s sensory experience. While traditional models focus on mechanical triggers like ductal hypertension and calcifications, many patients with advanced glandular burnout are asymptomatic, whereas those with minimal change disease may suffer from intractable pain [14]. This indicates that the pain phenotype often transitions from peripheral nociception to a centralized syndrome characterized by widespread hyperalgesia and impaired endogenous pain modulation. Consequently, the structural pathology visible on MRI or CT may represent only the initial trigger, while the current pain state is driven by stabilized neuroplastic changes in the central nervous system [28].

Chronic pain in CP is characterized by a definitive neuroplastic shift from peripheral nociception to a centralized pain state, mediated by central sensitization and the collapse of descending modulatory pathways. It involves the activation of NMDA receptors and the upregulation of voltage-gated calcium channels, which increase neuronal excitability and expand the receptive field. This spinal hyperexcitability is exacerbated by the failure of the descending inhibitory system, specifically the periaqueductal gray-rostral ventromedial medulla (PAG-RVM) axis. The resulting impairment in conditioned pain modulation (CPM) renders the central nervous system hyper-responsive to minimal peripheral input, explaining the clinical manifestations of allodynia and hyperalgesia that often persist independently of the initial glandular pathology.

## 3. Effect of Psychosocial Factors and Anxiety on CP

There is a vicious circle of pain and anxiety in CP. It has been established that pain perception is adversely affected by anxiety, stress, and depression. These, in turn, have a negative effect on treatment compliance, sleep quality, and pain levels [26,29]. This generates long-term dysfunction of the neuromatrix due to the painful condition [27]. Overstimulation of the sympathetic nervous system increases plasma norepinephrine levels, leading to hyperalgesia and enhancing central pain-processing pathways. The molecular mechanism of psychosocially augmented pain in CP involves the sympathetic-medullary axis and the dysregulation of the monoaminergic descending system. Chronic stress and anxiety trigger a sustained surge in systemic norepinephrine, which binds to $\beta$-adrenergic receptors on pancreatic nociceptors, lowering their activation threshold and facilitating peripheral sensitization [30]. Centrally, the cognitive-affective distress associated with depression and catastrophizing drives the activation of NMDA receptors in the dorsal horn while simultaneously exhausting the pool of serotonin and norepinephrine in the descending inhibitory pathways [24,26].

**Figure 1:**
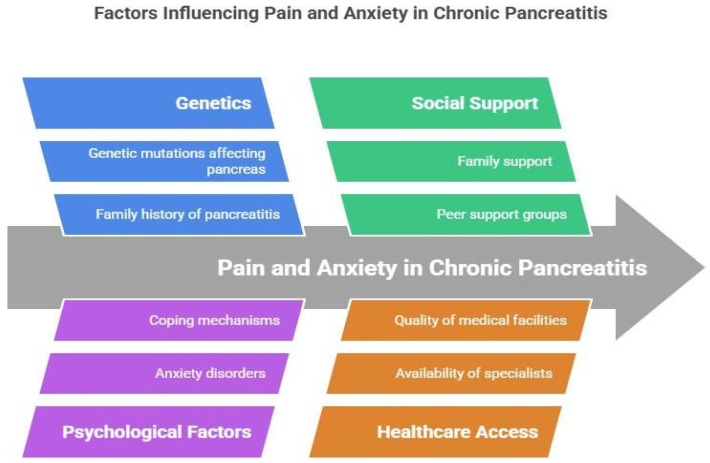
Various factors affecting pain and anxiety in chronic pancreatitis. The major factors are genetics, psychological factors, social support and healthcare access. The pathogenesis and anxiety of pain in chronic pancreatitis (CP) is modulated by a complex interplay of genetic, psychological, and systemic factors that dictate the transition from acute nociception to a chronic, centralized pain state.

To effectively manage this condition, analgesic drugs along with psychosocial support must be provided to the patients, including cognitive-behavioral therapy and support groups [26] (Figure 1). The psychological distress observed among CP patients is not just a result of their enduring pain but is an integral component of it. A high prevalence of anxiety, depression, and unhealthy coping styles exists among CP patients, and these psychological conditions are strongly correlated to their pain levels and reduced quality of life [12,13,31]. Changes in pain perception are mediated by central mechanisms influenced by emotional state. The anterior cingulate cortex, insula, and prefrontal areas have shown increased pain anticipation and catastrophizing, reduced pain thresholds, and are linked to heightened pain signaling. The continuous activation of the sympathetic nervous system (SNS) is associated with psychological distress. SNS activation causes an increase in catecholamine release, directly correlating with the excitability of nociceptors, establishing a physiological link through which anxiety can heighten pain perception or signaling [30]. This interaction maintains a self-perpetuating cycle wherein pain reinforces the level of anxiety, and the increased level of anxiety, through the system of neuroendocrine responses, enhances the overall level of pain perception. Quantitative sensory and intervention studies have provided supporting data for this proposed model. Patients with higher psychological burdens have greater abnormalities in pain modulation. These patients benefit from cognitive-behavioral interventions to improve outcomes in pain by changing coping skills and pain mechanisms, rather than modulating the pathology of the pancreas itself [26,32]. All these observations further emphasize the need to address both nociceptive input and the psychological factors that sustain central sensitization. Sexual dimorphism in CP pain is driven by estrogen-mediated modulation of the opioidergic system, which often results in lower mechanical pain thresholds and reduced conditioned pain modulation in women compared to men. This biological vulnerability is further exacerbated by higher reported rates of pain catastrophizing and stress-induced HPA-axis reactivity, which amplify the affective-motivational processing of pain and accelerate the transition toward a centralized pain phenotype.

## 4. Behavioral and Emotional Manifestations, and Impact on Physical Health

CP patients may suffer from sleep disorders, irritability, and fatigue, all of which may affect pain and anxiety [33]. Anxiety symptoms may lead to poor coping behaviors as well as high healthcare utilization [34]. Social withdrawal and decreased involvement in daily activities may increase levels of anxiety with additional emotional distress [31]. Anxiety may also elevate gastrointestinal motility, leading to increased symptoms of bloating and nausea [35]. In the rat immune system, chronic stress has been associated with increased susceptibility to infections and pancreatic damage; however, this has not been successfully validated in humans [36].

Behavioral and emotional changes are not limited to disturbances in the psyche but also to physical pain. For instance, sleep disturbances were seen to significantly decrease pain thresholds, heightening central sensitization. Similarly, fatigue and sedentary lifestyles further reduce the patient’s overall capacity to modulate pain, increasing pain levels during daily activities. Social withdrawal has been seen to play a role in the patient’s pain experience. Patients exhibiting higher levels of catastrophizing and the overall use of maladaptive coping mechanisms may exhibit heightened levels of pain, irrespective of the pathology in the pancreas. The psychological burden correlates with the quality-of-life impairment and the pain levels, as opposed to the radiological severity of the disease [12,33].

Anxiety-related changes in the gastrointestinal motility offer a further route through which visceral pain symptoms become enhanced. The slowing rate of gastric emptying, bloating, and nausea can all contribute to enhanced discomfort and the perception of pain, leading to further distress and the perseverance of the pain-anxiety cycle [35]. Behavioral symptoms of CP carry not only the consequence of pain but also contribute to the enhancement of pain perception.

## 5. Evaluation of Pain Development

A combined clinical, radiological, and laboratory approach to investigating the etiology of pain in CP should be utilized. The type, character, and associated symptoms of pain should be discovered through a comprehensive medical and clinical history. Pain measurement tools used to assess pain in CP patients include the Brief Pain Inventory (BPI), the Comprehensive Pain Assessment Tool (COMPAT), and Izbicki’s Pain Scale. With these tools, the severity of pain and its impact on quality of life can be measured to assess the efficacy of treatment methods [23,37]. Imaging studies such as CT, MRI, MRCP, and ERCP are performed to evaluate changes in pancreatic morphology, including dilatation or narrowing of the pancreatic ducts or pancreatic calcification [38]. Laboratory tests, including pancreatic function tests and biomarkers, help assess pancreatic function and inflammation [39]. Although conventional imaging and laboratory data are essential tools for assessing pancreatic structure and function, they rarely correlate with the severity of pain. Patients with minimal radiologic abnormalities may present with severe, debilitating pain, whereas others with extremes of calcification and abnormalities of the pancreatic duct may be relatively asymptomatic. Quantitative sensory testing (QST) is an important method to understand the development of pain in CP. Investigation into pain thresholds, i.e., levels of pain, at pancreatic and distant somatic sites has revealed generalized hyperalgesia and impaired pain modulation, supporting systemic alterations in sensory processing rather than pancreatic abnormalities alone [10,23]. The psychological evaluation of a CP patient is also crucial for an accurate assessment of pain. Evaluation of psychological elements such as anxiety, depression, poor coping, and sleep, through screening and assessment tools, provides important evidence for elements that may contribute to an enhanced perception of pain despite conventional therapies. Using a psychological assessment tool along with a pain scale allows practitioners to identify patients who present with predominant psychological and central effects, which can guide patient-specific treatment plans [26,40]. [AS1] [BA2] Pediatric pain assessment in CP requires developmentally tailored tools to accurately capture the subjective experience of younger patients who may lack the vocabulary for complex sensory descriptions. The Wong-Baker FACES Scale is the gold standard for children aged 3 and older to communicate intensity through visual distress cues, while the FLACC scale (Face, Legs, Activity, Cry, Consolability) provides a validated behavioral framework for non-verbal or younger children [26]. As children reach adolescence, the Visual Analog Scale (VAS) or Numerical Rating Scale (NRS) is used to quantify pain intensity, often supplemented by multidimensional tools such as the PedsQL to assess the broader psychosocial impact on quality of life.

## 6. Management Strategies

**Figure 2:**
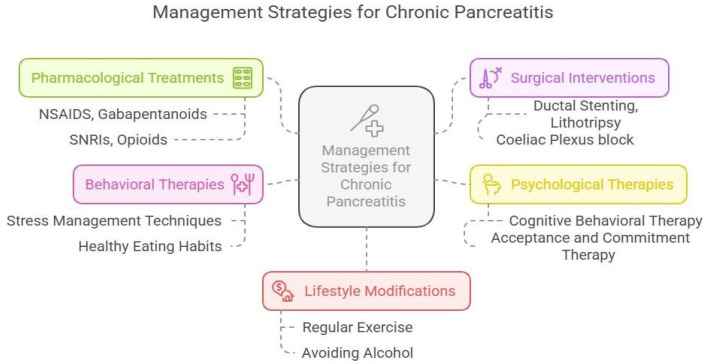
Management Strategies for Chronic Pancreatitis.

Management of CP involves a multidisciplinary approach that incorporates pharmacological [41,42], interventional and surgical [43,44], psychological and behavioral therapies [32,40], and lifestyle modifications [45,46,47], all of which are crucial for preventing disease progression and improving quality of life (Figure 2). Management of the CP pain phenotype requires a multimodal, biopsychosocial approach that targets both the peripheral organ and the sensitized nervous system. Beyond traditional analgesics, neuromodulators such as pregabalin or gabapentin are utilized to dampen the hyper-excitability of the central neuromatrix and address neuropathic pain.

Psychological interventions, specifically Cognitive-Behavioral Therapy (CBT) and mindfulness-based stress reduction, are critical for re-training the brain’s descending inhibitory pathways to counteract the effects of pain catastrophizing and depression. While endoscopic or surgical interventions may address structural triggers, their success often depends on early implementation before central sensitization becomes irreversible. For patients with a high psychosocial burden, interdisciplinary pain programs that integrate physical therapy, psychotherapy, and medical management provide the most effective framework for breaking the physiological cycle of anxiety and pain perception.

## 7. Chronic Pancreatitis in Children

CP is rarely diagnosed in children, and its epidemiology is unknown. The incidence of CP has been known to increase with age; adults have a 4 to 9-fold higher incidence than younger individuals. The International Study Group of Pediatric Pancreatitis: In search for a cure (INSPPIRE) consortium proposes that CP is commonly caused by genetic mutations. The most common genetic mutation that predisposes children to chronic pancreatitis is PRSS1, while mutations in SPINK1, CFTR, and CTRC are also known predispositions. Other risk factors include obstructive causes (e.g., pancreas divisum), autoimmune causes, and metabolic causes [48]. CP is a poorly understood childhood condition; many cases are due to genetic or hereditary causes.

The INSPPIRE consortium was created to characterize the etiology of acute recurrent pancreatitis (ARP) and chronic pancreatitis (CP) in children. This cohort demonstrated that 67% of children with CP had a genetic predisposition, with mutations in the PRSS1 gene accounting for more than half of these cases. Nearly one out of every three also presented with associated congenital obstruction, the majority having pancreatic divisum. These findings are consistent with single-center cohorts of various ethnicities, including Korean, Italian, Polish, and American populations, in which there was again a high incidence of PRSS1, SPINK1, and CFTR mutations in ARP and CP in children. This pattern differs from adult cohorts, where environmental factors play a prominent role. The North American Pancreas Study-2 (NAPS2) identified alcohol as the primary cause in nearly half of the adult cases, with tobacco use frequently observed in idiopathic pancreatitis [48].

Anatomical causes such as Pancreas Divisum and annular Pancreas also predispose patients to recurrent acute and chronic pancreatitis. Other rare causes of non-genetic and non-congenital causes of CP are trauma, medication side effects, and Sphincter of Oddi dysfunction.

### 7.1. Co-Presenting Symptoms

Pediatric patients with CP will generally present with pain in the epigastrium. This pain may radiate from the epigastrium to the back and may be aggravated by eating or drinking. Over the episode, the severity of pain may increase, become constant, and debilitating, which impairs the patient’s quality of life and warrants multiple hospital admissions. Other common symptoms of chronic pancreatitis include nausea, vomiting, weight loss, and fatty stools. Recurrent episodes of chronic pancreatitis pain in children lead to repeated hospitalizations, use of potent pain medications (i.e., opioids and narcotics), and missed school days. Due to the chronic nature of the disease, patients may also report functional pancreatic enzyme insufficiency and/or type 3c pancreatic diabetes [49].

Pediatric patients with chronic pancreatitis may show signs of abdominal discomfort and often limit movement due to pain. In some cases, the diagnosis is made without a documented history of prior acute pancreatitis. Mornville et al. also emphasized that patients with cystic fibrosis-related classic exocrine pancreatic insufficiency should not be diagnosed with CP [50].

### 7.2. Management Options

The goal of medical therapy in chronic pancreatic patients is adequate pain control and enzyme replacement to improve the quality of life. In pediatric CP, both medical and surgical interventions are available. Medical management is the first-line therapy, along with endoscopic procedures [51]. Medical therapy usually provides symptomatic relief along with treating the complications associated with chronic pancreatitis.

In cases of exocrine pancreatic insufficiency, pancreatic enzyme replacement therapy can help with the digestion and absorption of nutrients in the intestines. Exogenous enzyme therapy may also help suppress endogenous pancreatic enzyme secretion, thereby limiting inflammation and associated pain. These therapies have been associated with reduced pain levels, prompting studies to understand this phenomenon and achieve more reliable results. The leading hypothesis for this mechanism is the theory of ductal and interstitial hypertension. This theory suggests that the increase in pancreatic secretions is associated with increased pressure in the pancreatic duct and interstitium, with decreased distensibility of the damaged parenchyma, thereby increasing pressure and decreasing pain. Dietary modifications include a low-fat diet and avoidance of oral intake, with nutrition provided via nasojejunal or gastrojejunal feeding tubes [52].

In severe CP, invasive therapies may alleviate symptoms. For example, endoscopic interventions and pancreatic drainage procedures have been reported to provide symptomatic relief. But Bondoc et al. reported limited use of these invasive techniques to provide durable, prolonged pain control and symptomatic treatment in CP patients [53].

### 7.3. Advanced Treatment Option for Debilitating Pancreatitis: Total Pancreatectomy with Islet Cell Autotransplantation (TPIAT)

While various medical therapies aim to reduce pain and slow progression, surgical intervention becomes necessary in cases of refractory pain or suspicion of pancreatic cancer [54]. For CP, a total or near-total pancreatectomy (TP), removing 95% or more of the pancreas, is often considered the most effective surgical option for pain relief [55].

**Figure 3:**
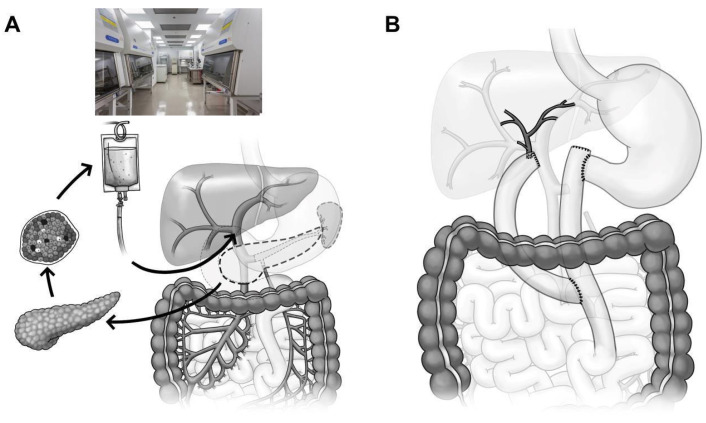
(**A**) Pancreatectomy followed by islet isolation in a clean room facility. Sequence of events to preserve beta cell mass in patients undergoing a total pancreatectomy for benign disease. The resected pancreas is dispersed by collagenase digestion followed by islet isolation. Autologous islets are then embolized to the patient’s liver by means of the portal vein (TPIAT). (**B**) Surgical technique for reconstruction following total pancreatectomy. Following total pancreatectomy and near-total duodenectomy (pylorus is preserved), the bile duct drainage is reconstructed via Roux-en-Y hepaticojejunostomy or choledochojejunostomy, and the alimentary tract is reconstructed via Roux-en-Y duodenojejunostomy.

An important complication of TP is iatrogenic diabetes, which results from the destruction of insulin-producing islets. This complication, however, can be avoided or minimized by performing simultaneous Islet Cell Auto Transplantation (IAT), which involves the infusion of islets into the portal vein. This serves to replace the islets, separated from the patient’s own pancreas, into the liver to produce endogenous insulin to prevent diabetes [56,57] (Figure 3).

Even though TP is a common surgical practice, IAT is not routinely performed because islet isolation laboratories are not readily available. The isolation of human islets is a highly complex and time-sensitive procedure. It requires highly trained and skilled personnel. The outcome of the islet isolation procedure is to provide the highest yield for auto transplantation. The islet isolation procedure involves organizing and preparing the required materials for pancreas trimming, pancreatic ductal cannulation, distension with the collagenase and protease enzyme mix, pancreas digestion, cell recombination, purification of the islets (if required), and transplant product packaging [58].

Total Pancreatectomy with Islet Auto Transplantation (TPIAT) offers a superior clinical trajectory compared to Total Pancreatectomy (TP) alone by targeting both the mechanical source of pain and the metabolic consequences of organ removal. While both procedures aim to eliminate nociceptive input to facilitate narcotic independence, TPIAT achieves this in up to 80% of patients while avoiding the “brittle diabetes” associated with TP. Mechanistically, the preservation of islet function through Auto Transplantation significantly enhances long-term survival and Quality of Life (QoL) by providing glycemic stability, which in turn reduces the psychosocial burden and physical disability associated with total endocrine loss [59].

In the pediatric population, TPIAT is indicated primarily for children with hereditary, genetic (e.g., PRSS1mutations), or idiopathic chronic pancreatitis who suffer from intractable pain and impaired quality of life despite exhaustive medical and endoscopic management. Clinical outcomes in children are generally superior to those of adult cohorts, with approximately 90% of pediatric patients experiencing significant pain relief and over 70% achieving complete narcotic independence within the first-year post-surgery. Furthermore, children demonstrate higher rates of insulin independence, often exceeding 50% at five years, due to a more robust islet mass and greater physiological resilience [60].

Despite these benefits, the procedure has significant limitations, including the risk of islet graft failure over time, the surgical complexity of the reconstruction, and the lifelong requirement for pancreatic enzyme replacement therapy. There is also the potential for post-operative gastroparesis or small bowel obstruction, which can complicate nutritional status in growing children. Furthermore, while TPIAT mitigates peripheral sources of pain, it cannot always reverse pre-existing central sensitization if surgery is performed too late in the disease course, necessitating continued multidisciplinary psychosocial support to manage the central pain phenotype [59,60].

## 8. Advancing SDGs Through Targeted Research

### 8.1. Rationale for Research

The pain experienced by patients with CP is chronic, debilitating, and creates emotional problems such as anxiety. The overall effect of these factors is significant to a patient’s quality of life, and this is quantified by mental and social factors or by the utilization of healthcare services [61]. The causes of CP differ between developed and developing countries. In developing countries, malnutrition and tropical pancreatitis are prominent causes of CP, while in developed countries, alcohol usage and genetic mutations are considered the main causes. Around the world, developed countries have more facilities, such as pain management and mental health experts, while in developing countries, due to inadequate facilities, pain management may be inadequate [62]. Pain and anxiety levels may be influenced by socioeconomic disparities. The ease of access to health facilities for CP treatment may differ by socioeconomic status and geographical location, among other factors [63,64]. Patients from different geographic locations may experience differences in treatment outcomes [61]. The evaluation of pain severity may also differ across ethnic groups [62,63]. It is important to consider the need for further research to determine the relationship between pain and anxiety for patients with a CP diagnosis. The clinical epidemiology of CP reveals a profound socioeconomic gradient, with low socioeconomic status a potent predictor of both disease incidence and phenotypic severity. Large-scale population studies demonstrate that individuals in the lowest income quintiles have a two- to threefold higher hazard ratio for CP, driven by the cumulative effects of environmental stressors, nutritional deficiencies, and restricted access to early diagnostic interventions.

The global burden of CP reflects a stark divide in healthcare infrastructure; while developed nations utilize multidisciplinary teams and advanced neuromodulation, developing regions often lack the facilities to manage complex pain, leading to inadequate treatment and a stabilized, centralized pain phenotype. Addressing these disparities requires a targeted research focus on the relationship between socioeconomic status and healthcare accessibility to ensure that life-altering interventions, such as TPIAT, do not remain restricted by geographical or financial barriers.

### 8.2. Call for Targeted Research

Targeted research is essential to uncover the relationship between pain and anxiety in CP. Since each component might exacerbate the condition, identifying different risk and contributing factors remains a priority. It may involve genetic, lifestyle, and environmental components. Establishing the relation between pain and anxiety will be critical in devising targeted, holistic interventions to improve the quality of life among people living with CP. This research would ultimately aid in developing a holistic treatment model. In-depth research across various geographical regions must be conducted to gather information on different populations and create patient-specific care plans.

Many current research endeavors seek to improve islet cell transplantation to enhance yield in total pancreatectomy with islet cell auto transplantation (TPIAT). Improvements in islet isolation techniques performed in a clean room under cGMP conditions have been investigated to increase the yield of isolated islets. Different strategies have been developed to increase islet cell yield by converting non-endocrine pancreatic cells into beta cells. Genomic and epigenetic changes in acinar cells, duct cells, and other pancreatic cells have been proposed as potential approaches to increase the number of beta cells for transplantation.

Future progress in chronic pancreatitis management depends on bridging critical evidence gaps through the discovery of validated biomarkers for central sensitization and the execution of longitudinal studies that track the co-evolution of pain and anxiety. Furthermore, large-scale intervention trials are required to evaluate integrated models that combine cellular advancements, like enhanced islet yield for TPIAT, with early-stage psychological and neuromodulatory therapies to achieve a truly holistic recovery.

### 8.3. Research Focus Aligning to Sustainable Development Goals (SDGs)

**Figure 4:**
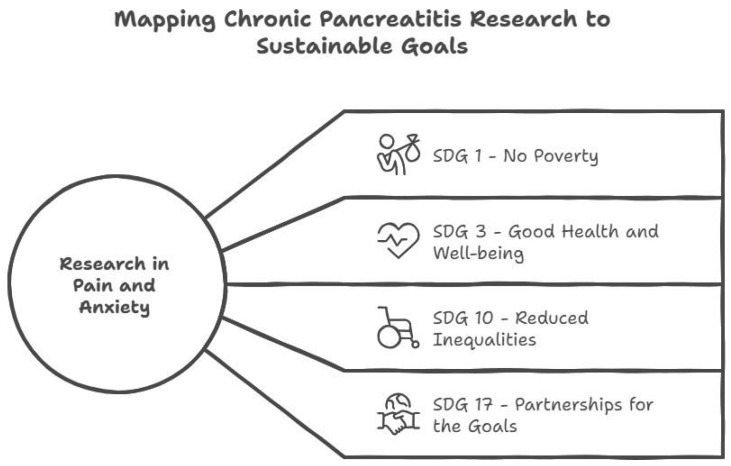
Mapping Chronic Pancreatitis Research to Sustainable Development Goals.

Studies should implement the SDGs, such as SDG 3 (Good Health and Well-being), in which research on CP pain and anxiety directly contributes to improving health outcomes and promoting well-being. SDG 1 (No Poverty) can be implemented for chronic illnesses, like CP, as they can create a significant financial burden due to medical expenses and lost productivity [64]. Research can help identify the socioeconomic factors contributing to CP risk and severity, and aid in developing cost-effective interventions. Research can advocate for the benefits of policies that provide financial support to CP patients, such as existing government programs [AS1] [BA2] (e.g., the Chief Minister’s Comprehensive Health Insurance Scheme and PM-JAY). These schemes help reduce inequality (SDG 10) by providing access to health care and treatment facilities for people with CP. It is essential to maintain a partnership among healthcare providers, researchers, and policymakers to effectively address the complexities of CP (SDG 17). This partnership helps us share existing knowledge, resources, and best practices to effectively manage CP patients. By linking our research to the SDGs, studies of the burden of pain and anxiety in CP aim to improve global health for all (Figure 4). Aligning CP research in India with the UN Sustainable Development Goals (SDGs) ensures that medical advancements translate into equitable health outcomes for the nation’s most vulnerable. By leveraging frameworks such as SDG 1 (No Poverty) and SDG 10 (Reduced Inequalities), research can advocate for the strategic expansion of government funding.

## 9. Conclusions

The development of pain in CP is a paradigm for the complex integrative action of central and peripheral mechanisms, which in turn are modulated by psychosocial and comorbid state factors. Pain in CP normally begins as intermittent pain but proceeds to chronic pain with a neuropathic and ischemic nature. Pain tends to increase with anxiety or psychological states. Understanding these mechanisms will help identify the most appropriate therapeutic regimens for this disease.

Targeted research is considered an essential aspect to gain a better understanding of the association between pain and anxiety associated with CP to find appropriate strategies to enhance the quality of life for the patients, especially considering the contrast between the developing and developed countries. To strengthen the clinical impact and provide a roadmap for future investigation, the take-home messages and research priorities should emphasize the neurobiological transition of the disease and the urgent need for biopsychosocial stratification.

## Data Availability

No new data was created for this study.

## Abbreviations

The following abbreviations are used in this manuscript:

CP	Chronic Pancreatitis
TPIAT	Total pancreatectomy with islet auto transplantation
CFTR	Cystic fibrosis transmembrane conductance regulator
PRSS1	Cationic trypsinogen
SPINK1	Pancreatic secretory trypsin inhibitor
CTRC	Chymotrypsin C
CPA1	Carboxypeptidase 1
SDG	Sustainable development goals
SHEs	Severe hypoglycemic events
